# Ten Minutes Advice Respecting the Human Teeth

**Published:** 1852-04

**Authors:** 


*Ten Minutes Advice respecting the Human Teeth.* By Horace Norton, M. D. Watertown, Wisconsin: Milwauke, 1851.

A book from Wisconsin! The wonder of the grateful man who had unexpectedly got a dinner from a miser, broke out in a poetical ejaculation,

> "In the midst of famine, we have found relief,
> And seen the wonder of a chine of beef!"

Very similar was our wonder to see a book from the wilderness. But it seems that it is a wilderness no longer. A man need not sleep like Rip Van Winkle, to get behind his age. He need only sleep for a single night, to find his notions wrong. A book from Wisconsin! and not a book of travels nor a description of wild cats, but about the teeth! We wish to see nature in her freshness before we die, but we must hasten or there will be no nature left to see.

The little book before us contains much excellent advice, and has less the appearance of an advertisement than most similar publications. We thank the author for the copy he has sent us.

